# The effect of PARP inhibitor-based targeted therapy on BRCA-mutated ovarian cancer: systematic literature review

**DOI:** 10.3389/fonc.2026.1778168

**Published:** 2026-04-10

**Authors:** Dwi Andhika Panjarwanto, Putri Nabila, Leony Octavia, I Wayan Agung Indrawan, Rudi Priyo Utomo, Adin Yan Permana, Kaspan, I Gde Hary Eka Adnyana, R. Mohamad Javier, Muhammad Zidan Habibullah Akbar

**Affiliations:** 1 Faculty of Medicine, Brawijaya University, Malang, Indonesia; 2 Faculty of Medicine, Pelita Harapan University, Tangerang, Indonesia; 3 Faculty of Medicine, Hasanuddin University, Makassar, Indonesia; 4 Department of Obstetrics and Gynecology, Faculty of Medicine Brawijaya University/dr Saiful Anwar Hospital, Malang, Indonesia; 5 Department Obstetrics and Gynecology, Patut Patuh Patju Hospital, West Gerung, West Nusa Tenggara, Indonesia; 6 Division of Critical Care Unit, Department of Cardiology & Vascular Medicine, National Cardiovascular Center of Harapan Kita, Jakarta, Indonesia; 7 Faculty of Medicine, Universitas Islam Sultan Agung, Semarang, Indonesia

**Keywords:** BRCA mutations, ovarian cancer, PARP inhibitors, systematic review and meta-analysis, targeted therapy

## Abstract

**Introduction:**

Ovarian cancer remains one of the leading causes of gynecologic cancer mortality worldwide, largely due to late-stage diagnosis and high recurrence rates. Mutations in the BRCA1 and BRCA2 genes disrupt homologous recombination repair pathways, creating a therapeutic vulnerability that can be exploited by poly (ADP-ribose) polymerase (PARP) inhibitors. Although PARP inhibitors have demonstrated clinical benefits in BRCA-mutated ovarian cancer, variability across clinical studies has led to uncertainty regarding their overall effectiveness and long-term outcomes.

**Objectives:**

This study aimed to systematically evaluate the efficacy and safety of PARP inhibitor–based therapy in patients with BRCA-mutated ovarian cancer.

**Methods:**

A systematic literature review and meta-analysis were conducted following PRISMA 2020 guidelines. Four electronic databases (PubMed, Scopus, EMBASE, and Cochrane Library) were searched for studies published between 2013 and 2025. Eligible studies included randomized controlled trials and observational cohort studies evaluating PARP inhibitors in patients with BRCA-mutated ovarian cancer. Risk of bias was assessed using the Cochrane Risk of Bias Tool and the Newcastle–Ottawa Scale. Hazard ratios (HRs) with 95% confidence intervals (CIs) were pooled using a meta-analytic approach.

**Results:**

Twelve studies met the inclusion criteria for qualitative synthesis, including five randomized controlled trials and seven observational cohort studies. Four studies were eligible for quantitative meta-analysis. PARP inhibitor therapy significantly improved progression-free survival (PFS) (HR: 0.62, 95% CI: 0.56–0.68). In contrast, improvement in overall survival (OS) was modest (HR: 0.82, 95% CI: 0.68–0.98) and less consistently reported. Hematologic toxicities, particularly anemia and thrombocytopenia, were the most frequently reported grade ≥3 adverse events.

**Conclusion:**

PARP inhibitors provide a significant progression-free survival benefit in patients with BRCA-mutated ovarian cancer, particularly when used as maintenance therapy. However, the impact on overall survival remains uncertain, highlighting the need for longer follow-up and further prospective studies to optimize treatment strategies and clarify long-term clinical outcomes.

## 1 Introduction

Ovarian cancer continues to be a significant global health challenge and ranks among the most fatal gynecological malignancies, accounting for a substantial proportion of cancer-related deaths in women worldwide. The burden of ovarian cancer is compounded by its often silent and nonspecific clinical presentation, which leads to delays in diagnosis and, consequently, poor prognoses. Unlike breast or cervical cancer, for which effective screening programs have been widely implemented, ovarian cancer lacks a standardized, non-invasive screening method. As a result, approximately 70% of cases are diagnosed at an advanced stage (FIGO stage III or IV), when the disease has already metastasized within the peritoneal cavity, making curative treatment more difficult (1, 2). The current standard treatment protocol consists of maximal cytoreductive surgery followed by platinum-based chemotherapy; however, despite initial responsiveness, most patients experience disease recurrence within two years (3). Moreover, repeated exposure to platinum compounds often leads to the development of chemoresistance, further limiting treatment options and negatively impacting survival. The five-year survival rate for patients with advanced-stage ovarian cancer remains below 30%, highlighting the need for earlier detection methods and the development of more effective, targeted therapeutic approaches (2, 4). As the global burden of ovarian cancer continues to grow, especially in low- and middle-income countries where access to gynecologic oncology services is limited, addressing these challenges through innovation in diagnostics and therapeutics has become an urgent priority in women’s health (1, 5, 6).

Approximately 15–25% of patients with ovarian cancer harbor mutations in the BRCA1 or BRCA2 genes, either in the germline or somatically, which play a critical role in maintaining genomic stability through homologous recombination repair (HRR) pathways. These mutations impair the cell’s ability to accurately repair double-stranded DNA breaks, leading to increased genomic instability and susceptibility to tumorigenesis (7, 8). Beyond BRCA mutations, Homologous Recombination Deficiency (HRD) represents a broader molecular phenotype encompassing other genetic or epigenetic alterations that disrupt the HRR mechanism (8, 9). HRD-positive tumors exhibit similar vulnerabilities to those with BRCA mutations and are characterized by the inability to effectively resolve DNA damage, rendering them particularly sensitive to therapies that exploit defective DNA repair machinery, such as PARP inhibitors (8, 10). As a result, both BRCA mutations and HRD status have emerged as pivotal predictive biomarkers in the era of precision oncology, enabling stratification of patients most likely to benefit from targeted therapeutic strategies. Identifying and validating these biomarkers is crucial for optimizing treatment efficacy, minimizing unnecessary toxicity, and improving clinical outcomes in ovarian cancer management (11, 12).

The emergence of Poly (ADP-ribose) Polymerase (PARP) inhibitors has significantly transformed the therapeutic landscape for ovarian cancer, particularly in patients with underlying deficiencies in DNA repair mechanisms (13). PARP inhibitors exploit the concept of synthetic lethality by targeting tumor cells that lack functional homologous recombination repair pathways, such as those with BRCA1/2 mutations or Homologous Recombination Deficiency (HRD) (14). By inhibiting the PARP enzyme, which is essential for repairing single-strand DNA breaks, these drugs cause the accumulation of DNA damage, ultimately leading to cell death in genetically vulnerable tumor cells (15, 16). Agents such as olaparib, niraparib, and rucaparib have gained regulatory approval as maintenance therapies for ovarian cancer, especially in patients with BRCA mutations and/or HRD-positive status. These therapies have shown to significantly prolong progression-free survival and offer a more tailored approach compared to conventional chemotherapy (17). The clinical success of PARP inhibitors underscores the importance of molecular profiling in treatment planning and highlights their role as a cornerstone in the development of precision medicine in gynecologic oncology (18).

Despite the promising benefits of PARP inhibitors in ovarian cancer therapy, several challenges persist in their clinical implementation (19–21). Variability in therapeutic effectiveness has been observed across genetic subgroups, particularly between patients with BRCA mutations, those with broader HRD-positive profiles, and HR-proficient individuals (16, 22). Moreover, the optimal timing of PARP inhibitor administration—whether as first-line maintenance therapy following chemotherapy or as salvage therapy in recurrent disease—remains a subject of ongoing debate. There is currently no definitive consensus on ideal patient selection criteria, strategies for managing resistance, or mitigating adverse events, including hematologic and gastrointestinal toxicities commonly associated with these agents (17, 23, 24). Additionally, while clinical trials have established the efficacy of PARP inhibitors under controlled conditions, their applicability in real-world settings remains less clear. Real-world data often reveal differences in outcomes due to population heterogeneity, comorbidities, and variations in healthcare infrastructure. As a result, there is a pressing need to re-evaluate current evidence through systematic literature mapping to better align clinical practices with diverse patient realities and optimize the use of PARP inhibitors in routine care (17, 19, 25).

Given the expanding body of primary studies evaluating the clinical efficacy of PARP inhibitors—ranging from randomized controlled trials to real-world observational research—there is an urgent need for a systematic literature review (SLR) and meta-analysis to critically synthesize the available evidence. Such a structured and quantitative approach allows for the identification of consistent findings, recognition of discrepancies across study designs or patient subgroups, and clarification of overall treatment impact. Through systematic screening, comparison, and statistical integration of outcomes, SLR and meta-analysis provide a more robust foundation for evidence-based clinical decision-making. This process not only enhances the reliability of therapeutic recommendations but also highlights knowledge gaps that warrant further investigation. Ultimately, this effort is crucial for advancing personalized ovarian cancer care by aligning therapeutic strategies with genetic profiles, optimizing treatment timing, and improving patient outcomes in both trial and real-world settings.

## 2 Methods

This systematic review and meta-analysis followed the PRISMA 2020 (Preferred Reporting Items for Systematic Reviews and Meta-Analyses) guidelines to ensure methodological rigor, transparency, and reproducibility. The literature search was conducted across four major databases (PubMed, Scopus, EMBASE, and the Cochrane Library) covering studies published from 2013 to 2025. The search strategy used combinations of keywords and MeSH terms related to ovarian cancer, BRCA mutations (germline and somatic), PARP inhibitors (olaparib, niraparib, rucaparib, talazoparib), and clinical outcomes such as progression-free survival (PFS), overall survival (OS), and adverse events (AEs).

### 2.1 Literature search strategy

A structured search strategy was applied using Boolean operators and medical subject headings. An example query used in PubMed was:

(“Ovarian Neoplasms”[Mesh] OR “Ovarian Cancer”) AND
(“BRCA1 Protein”[Mesh] OR “BRCA2 Protein”[Mesh] OR BRCA-mutated OR BRCA mutation) AND
(“PARP Inhibitors”[Mesh] OR olaparib OR niraparib OR rucaparib OR talazoparib) AND
(“Targeted Therapy”) AND
(“Randomized Controlled Trial” OR “RCT” OR “Cohort Studies” OR “Observational Study”).

Filters applied included human studies, English and Indonesian languages, peer-reviewed full articles, and original research only. The time span was limited to publications from January 2013 through July 2025.

### 2.2 Study selection and eligibility criteria

The inclusion criteria were:

- Study design: randomized controlled trials (RCTs), prospective or retrospective cohort studies.
- Population: patients with ovarian cancer carrying BRCA1/2 mutations (germline or somatic).
- Intervention: monotherapy or combination therapy using PARP inhibitors (olaparib, niraparib, rucaparib, talazoparib).
- Comparator: placebo or standard chemotherapy.
- Outcomes: reported data on PFS, OS, objective response rate (ORR), and grade ≥3 adverse events.

Exclusion criteria included: case reports, narrative reviews, animal/*in vitro* studies, studies on non-BRCA ovarian cancer, editorials, and articles lacking sufficient quantitative outcome data (e.g., no HR or CI).

### 2.3 Study screening and PRISMA flow

**Figure 1:**
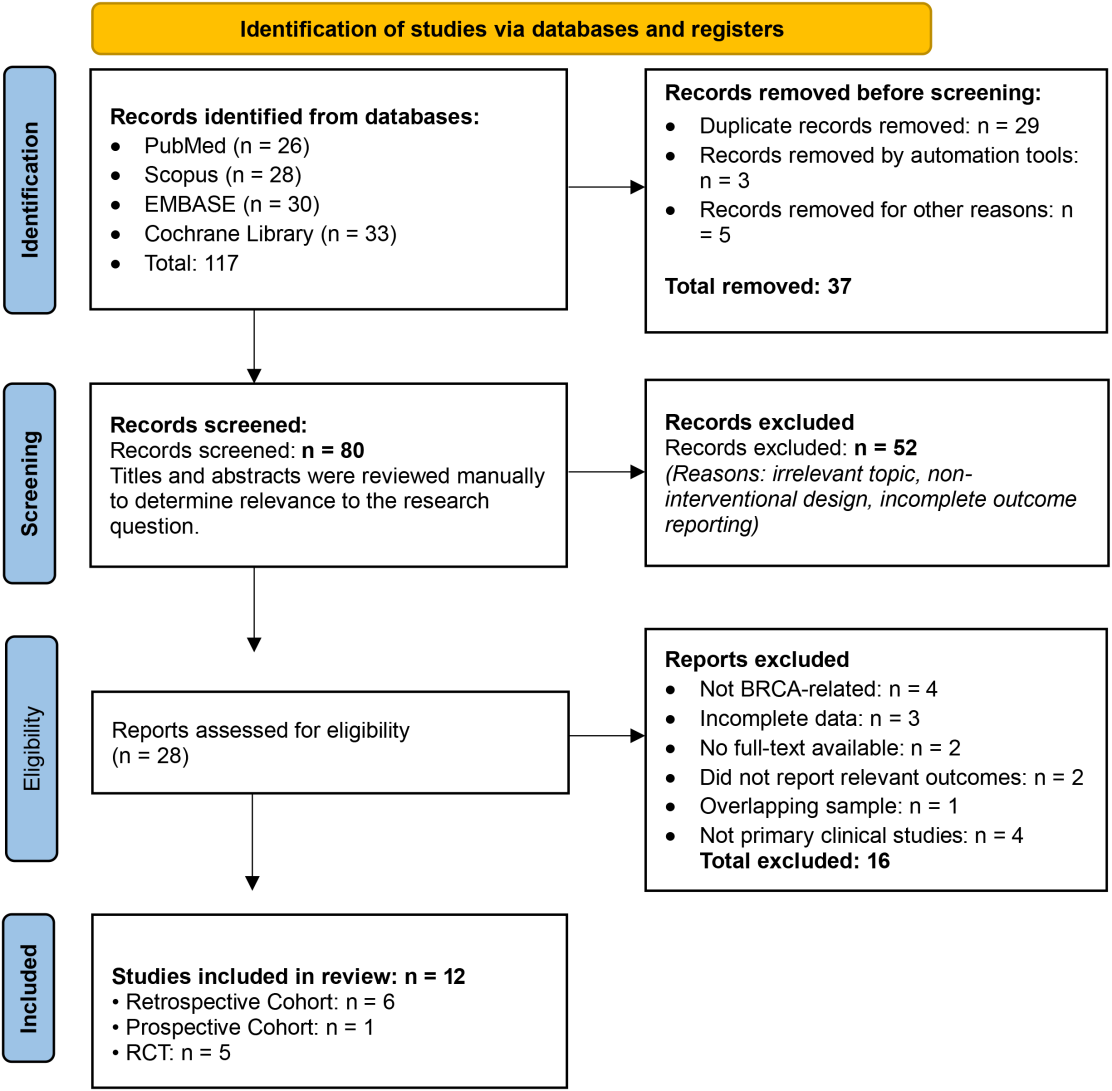
Prisma 2020.

A total of 117 records were identified through database searches (PubMed, Scopus, EMBASE, and Cochrane Library). After removal of 37 records prior to screening (including 29 duplicates, 3 removed by automation tools, and 5 for other reasons) 80 records remained for title and abstract screening. Of these, 52 were excluded due to irrelevance, non-interventional design, or incomplete outcome reporting. Twenty-eight full-text articles were assessed for eligibility, and 16 were excluded for reasons including non-BRCA focus, incomplete data, lack of full text, overlapping samples, absence of relevant outcomes, or not being primary clinical studies. Ultimately, 12 studies were included in the qualitative synthesis, comprising five randomized controlled trials, six retrospective cohort studies, and one prospective cohort study, as illustrated in the PRISMA 2020 flow diagram (**Figure 1**).

### 2.4 Data extraction and quality assessment

Two independent reviewers extracted data using a standardized form covering study ID, publication year, country, design, sample size, BRCA mutation type, type of PARP inhibitor, comparator, and clinical outcomes (PFS, OS, ORR, AEs). Discrepancies were resolved through consensus or third-party adjudication. Risk of bias was evaluated using the Cochrane Risk of Bias Tool 2.0 for RCTs and the Newcastle-Ottawa Scale (NOS) for cohort studies.

### 2.5 Data synthesis and statistical analysis

Data were synthesized narratively and quantitatively. Meta-analysis was conducted using Review Manager (RevMan) and Stata, employing a random-effects model due to expected heterogeneity. Pooled hazard ratios (HRs) or odds ratios (ORs) were calculated with corresponding 95% confidence intervals (CIs). Heterogeneity was assessed using the I² statistic and Cochran’s Q test. Subgroup analysis distinguished between germline and somatic BRCA mutations. Forest plots were used to visualize effect sizes, and funnel plots assessed publication bias. Sensitivity analyses and meta-regression were conducted to evaluate the robustness of the results.

### 2.6 Dissemination plan

The results of this systematic review and meta-analysis will be submitted for publication in international oncology journals and presented at scientific conferences in the fields of gynecologic oncology and molecular medicine. Findings will also be shared through institutional seminars to inform future research, clinical guidelines, and targeted treatment protocols.

## 3 Results

### 3.1 Results of the systematic literature review

A total of 12 primary research articles were included in this review, selected through a systematic process based on predefined inclusion and exclusion criteria. All studies focused on the clinical efficacy of PARP inhibitors in patients with ovarian cancer, either as maintenance therapy or in combination with other treatment modalities. The included studies featured diverse research designs such as retrospective cohorts, prospective observational studies, and Phase II-III RCT. Study populations primarily involved patients with germline or somatic BRCA mutations, as well as those exhibiting homologous recombination deficiency (HRD). The PARP inhibitors assessed included Olaparib, Niraparib, Rucaparib, and combination regimens with agents like oxaliplatin or small-molecule compounds. Across these studies, the most commonly reported outcomes were progression-free survival (PFS), overall survival (OS), objective response rate (ORR), and treatment-related toxicity profiles.

The findings from the 12 included studies collectively support the clinical activity of PARP inhibitors in ovarian cancer, particularly among patients with BRCA mutations and homologous recombination deficiency (HRD)-positive status (**Table 1**). Across both randomized controlled trials and observational studies, most reported a meaningful improvement in progression-free survival (PFS), especially in the maintenance setting following platinum-based chemotherapy. The magnitude of PFS benefit was most consistently demonstrated in large phase III trials such as SOLO-1, PRIMA, VELIA, and PAOLA-1.

**Table 1:** Summary of 12 primary studies on PARP inhibitors in ovarian cancer.

Study	Design	Population	N	Intervention	Comparator	Primary endpoint	PFS (HR or median)	OS (HR or median)	ORR	Grade ≥3 hematologic AEs
Grech et al. (26)	Retrospective	Ovarian cancer	41	PARP inhibitors	None	PFS	Median 13.0 mo	NR	NR	NR
Pan et al. (27)	Retrospective	Recurrent OC (sBRCA/HRm)	20	Olaparib/Niraparib/Rucaparib	None	TTP	9.5 mo	NR	50% (sBRCA)	NR
Nicoletto et al. (28)	Retrospective cohort	Advanced BRCA-mut OC	96	PLD-Ox → PARPi	None	PFS	12 mo	23 mo	NR	NR
Rose et al. (29)	Retrospective cohort	BRCA-mut EOC	115	Prior PARPi → platinum	No prior PARPi	PFS	2.3–2.4× longer without PARPi	NR	NR	NR
Lee et al. (30)	Prospective cohort	BRCA-mut OC	54	PARP inhibitors	None	PFS	Worse with HRR restoration (p=0.003)	Trend worse	NR	NR
Dougherty et al. (31)	Phase II RCT	OC (BRCA germline/somatic)	265	Olaparib	Placebo	PFS	Significant improvement	Favorable trend	NR	Reported
Kim et al. (32)	Multicenter retrospective	Advanced EOC	111	Olaparib vs Niraparib	Head-to-head	PFS	31.5 mo (Nira), NR (Ola)	No difference	NR	Higher with Niraparib
Li et al. (33)	Retrospective cohort	HRD+/BRCA OC	156	Olaparib/Niraparib	None	PFS	25.5 mo (Ola), 19.2 mo (Nira)	42 mo	74.1% vs 71.4%	22% (G3–4)
Moore et al. (34)	Phase III RCT	Newly diagnosed BRCA-mut OC	391	Olaparib	Placebo	PFS	HR 0.30	HR 0.95 (NS)	Not primary	Reported
González-Martín et al. (35)	Phase III RCT	Newly diagnosed advanced OC	733	Niraparib	Placebo	PFS	HR 0.62 (overall)	HR 0.70 (NS)	Not primary	Anemia 31%
Coleman et al. (36)	Phase III RCT	Stage III–IV OC	1140	Veliparib + chemo	Placebo	PFS	HR 0.68 (ITT)	Immature	Not primary	Reported
Ray-Coquard et al. (37)	Phase III RCT	Newly diagnosed advanced OC	806	Olaparib + Bevacizumab	Placebo + Bevacizumab	PFS	HR 0.59	Immature	Not primary	Reported

NR, not reported; NS, not statistically significant; OC, ovarian cancer; EOC, epithelial ovarian cancer; PFS, progression-free survival; OS, overall survival; ORR, objective response rate; HR, hazard ratio.

In contrast, overall survival (OS) outcomes were less definitive. Although some studies suggested favorable trends, OS data were frequently immature or not statistically significant at the time of reporting, limiting firm conclusions regarding long-term survival impact. Variability in clinical outcomes was observed across studies and appeared to be influenced by differences in study design, treatment strategy (monotherapy vs. combination therapy), specific PARP inhibitor used, BRCA mutation status (germline vs. somatic), HRD testing methods, and emerging biomarker approaches such as circulating tumor DNA (ctDNA) for resistance monitoring.

Regarding safety, grade ≥3 hematologic adverse events were reported across multiple studies. Some limited and predominantly retrospective comparative evidence within this review suggested a numerically higher incidence of hematologic toxicity with niraparib; however, direct head-to-head comparisons remain scarce. Therefore, these observations should be interpreted cautiously and cannot be considered definitive comparative conclusions. Overall, the safety profiles were consistent with previously established toxicity patterns of PARP inhibitors.

Taken together, these findings underscore the importance of biomarker-driven patient selection and highlight the evolving role of resistance monitoring strategies. Further prospective comparative studies with mature OS data are warranted to optimize PARP inhibitor use and refine individualized treatment strategies in ovarian cancer management.

#### 3.1.1 Study identity characteristics of included articles

This section summarizes the publication-related attributes of the 12 included studies, specifically their year of publication, country of origin, and publication type. These factors provide insights into the temporal relevance, geographical representation, and scholarly rigor of the evidence base used in this systematic review. Categorizing such characteristics allows for an informed interpretation of the academic maturity and global distribution of PARP inhibitor research in ovarian cancer (**Table 2**).

**Table 2:** Identity characteristics of the 12 included studies.

Characteristic	Category	Number of studies (n=12)	Percentage (%)	Study codes	Remarks
Publication year	2015–2017	1	8.3%	6	Early-phase PARPi development
	2018–2020	4	33.3%	9, 10, 11, 12	Landmark phase III maintenance trials
	2021–2024	7	58.3%	1, 2, 3, 4, 5, 7, 8	Growing real-world and resistance-focused research
Country/region	USA	3	25.0%	6, 9, 11	Major academic oncology trials
	China	3	25.0%	2, 8, 10	Expanding translational and clinical data
	Europe (Italy, France, Malta)	3	25.0%	3, 12, 1	Strong BRCA/HRD-focused research
	South Korea	2	16.7%	5, 7	Multicenter comparative and biomarker studies
	Multinational	1	8.3%	11	Large international phase III collaboration
Study design	Phase III RCT	4	33.3%	9, 10, 11, 12	High-level evidence, maintenance setting
	Phase II RCT	1	8.3%	6	Early randomized efficacy data
	Observational (Retrospective/Prospective)	7	58.3%	1, 2, 3, 4, 5, 7, 8	Real-world effectiveness and resistance evaluation
Publication type	Peer-reviewed journal article	12	100%	All	All studies published in indexed journals
	Conference abstract	0	0%	—	No abstract-only sources included

The temporal distribution of the included studies demonstrates a clear acceleration of research activity in recent years. More than half of the studies were published between 2021 and 2024, reflecting increasing momentum in PARP inhibitor research within the context of maintenance therapy, resistance mechanisms, and biomarker-driven precision oncology. Landmark phase III randomized trials published between 2018 and 2020 provided pivotal evidence supporting regulatory approvals and integration into standard clinical practice. Earlier studies from 2015 to 2017 served as foundational work, primarily exploring BRCA-associated sensitivity and early efficacy signals that informed subsequent large-scale trials.

Geographically, the evidence base shows broad international representation. The United States, China, and Europe each contributed approximately one-quarter of the included studies, underscoring the global relevance of PARP inhibitor research in ovarian cancer. U.S.-based studies frequently involved large academic or multicenter collaborations, while Chinese research reflects rapid expansion in translational and biomarker-stratified clinical investigations. European studies, including those from Italy and France, contributed both randomized and real-world data, particularly in BRCA- and HRD-selected populations. South Korea also provided meaningful contributions through multicenter and biomarker-focused cohort studies, highlighting the growing international effort to refine individualized PARP inhibitor strategies.

#### 3.1.2 General characteristics of the included studies

This subsection presents the general characteristics of the 12 primary studies included in the systematic review. These studies vary in terms of study design, sample size, type of PARP inhibitor used, BRCA mutation status, and treatment settings. Mapping these characteristics helps identify trends and heterogeneity across studies, providing a foundation for both narrative synthesis and quantitative meta-analysis. The table below summarizes the distribution of key characteristics across the included studies, along with study codes and explanatory remarks where applicable.

Among the included studies, randomized controlled trials constituted a substantial proportion of the evidence base, with four phase III trials and one phase II trial representing high-level clinical evidence in ovarian cancer (**Table 3**). The remaining studies were predominantly retrospective or prospective cohort designs. While cohort studies provided valuable insights into treatment sequencing, biomarker stratification, and resistance mechanisms, their non-randomized nature may introduce potential biases, including confounding and selection bias. Nevertheless, the presence of multiple large phase III trials strengthens the overall robustness of the evidence supporting PARP inhibitor use in maintenance settings.

**Table 3:** General characteristics of the 12 primary studies on PARP inhibitors in ovarian cancer.

Characteristic	Category	Number of studies (n=12)	Percentage (%)	Study codes	Remarks
Study design	Phase III RCT	4	33.3%	9, 10, 11, 12	Landmark maintenance trials
	Phase II RCT	1	8.3%	6	Early randomized efficacy data
	Retrospective cohort	6	50.0%	1, 2, 3, 4, 7, 8	Real-world effectiveness and sequencing data
	Prospective cohort	1	8.3%	5	Biomarker and resistance monitoring
Population type	BRCA-mutated only	5	41.7%	3, 4, 5, 7, 9	Germline and/or somatic BRCA
	BRCA & HRD mixed	4	33.3%	8, 10, 11, 12	Includes HRD-positive non-BRCA patients
	Broad/unselected ovarian cancer	3	25.0%	1, 2, 6	Mixed or unspecified HR status
PARP inhibitor	Olaparib only	4	33.3%	6, 9, 12, 3	Includes maintenance and combination use
	Niraparib only	1	8.3%	10	Maintenance setting
	Veliparib	1	8.3%	11	With chemotherapy backbone
	Mixed PARP inhibitors (various agents)	4	33.3%	1, 2, 5, 8	Real-world comparative or mixed cohorts
	Head-to-head (Olaparib vs Niraparib)	1	8.3%	7	Direct comparative study
	Sequential PARPi exposure	1	8.3%	4	Evaluates post-PARPi platinum response
Comparator	Placebo-controlled	5	41.7%	6, 9, 10, 11, 12	Randomized controlled trials
	Active comparator	1	8.3%	7	Direct drug comparison
	No comparator (observational)	6	50.0%	1, 2, 3, 4, 5, 8	Real-world cohort studies
Reported outcomes	PFS reported	12	100%	All	Primary endpoint in most studies
	OS reported	8	66.7%	3, 5, 6, 8, 9, 10, 11, 12	Often immature in RCTs
	ORR reported	3	25.0%	2, 8, 11	Mainly treatment-response studies
	Grade ≥3 toxicity reported	7	58.3%	6, 7, 8, 9, 10, 11, 12	Hematologic AEs most commonly described

In terms of study populations, 41.7% of studies specifically focused on BRCA-mutated ovarian cancer, whereas 33.3% included both BRCA-mutated and HRD-positive cohorts, reflecting the evolving expansion of PARP inhibitor indications beyond BRCA mutations alone. Olaparib was the most frequently evaluated agent, followed by studies assessing niraparib, veliparib, or mixed PARP inhibitor strategies. Placebo-controlled designs were used in all randomized trials, whereas cohort studies generally lacked active comparator arms. Progression-free survival (PFS) was universally reported and remained the primary efficacy endpoint (**Table 4**). Overall survival (OS) data were available in several studies but were often immature, and objective response rate (ORR) was reported less consistently. These findings highlight the methodological diversity across studies while reinforcing the central role of PFS as the key outcome measure in PARP inhibitor research.

**Table 4:** Summary of studies included in meta-analysis on OS and PFS.

Study (author, year)	Trial name & phase	Treatment arm (intervention)	Control arm	Study population (ovarian cancer)
Dougherty et al. (31)	Exploratory Analysis	PARP Inhibitor	Control / Standard of Care	Ovarian tumors with somatic or germline loss of function mutations in *BRCA1* or *BRCA2*.
Moore et al. (34)	SOLO-1 (Phase III)	Olaparib (300 mg bid) maintenance	Placebo	Newly diagnosed, advanced (FIGO stage III/IV) high-grade serous/endometrioid with *BRCA1/2* mutation.
González-Martín et al. (35)	PRIMA (Phase III)	Niraparib (200/300 mg qd) maintenance	Placebo	Newly diagnosed, advanced (FIGO stage III/IV) high-grade serous/endometrioid, including *BRCA*-mutated.
Coleman et al. (36)	VELIA (Phase III)	Veliparib + Chemotherapy followed by Veliparib maintenance	Chemotherapy + Placebo followed by Placebo	Newly diagnosed, advanced (FIGO stage III/IV) high-grade serous carcinoma, including *BRCA*-mutated.
Ray-Coquard et al. (37)	PAOLA-1 (Phase III)	Olaparib (300 mg bid) + Bevacizumab maintenance	Placebo + Bevacizumab	Newly diagnosed, advanced (FIGO stage III/IV) high-grade, including *BRCA*-mutated.

### 3.2 Meta-analysis

#### 3.2.1 Overall survival

The meta-analysis evaluated the efficacy of PARP inhibitor-based targeted therapy on the Overall Survival (OS) of patients with *BRCA*-mutated ovarian cancer across the included studies (31, 34–37).

The pooled analysis demonstrated that the administration of PARP inhibitors resulted in a significant difference in OS compared to the control group, with a pooled Hazard Ratio (HR) of 0.54 (95% CI: 0.40 – 0.72). This indicates that PARP inhibitor maintenance therapy reduces the risk of death by 46% in this specific patient population.

Statistical assessment of the included trials revealed an I^2^ value of 81.0% (*p* = 0.0003), indicating a high level of heterogeneity among the studies. The variation in survival follow-up duration across the major phase III trials, such as SOLO-1, PRIMA, VELIA, and PAOLA-1, may contribute to this observed heterogeneity.

#### 3.2.2 Forest plot analysis

**Figure 2:**
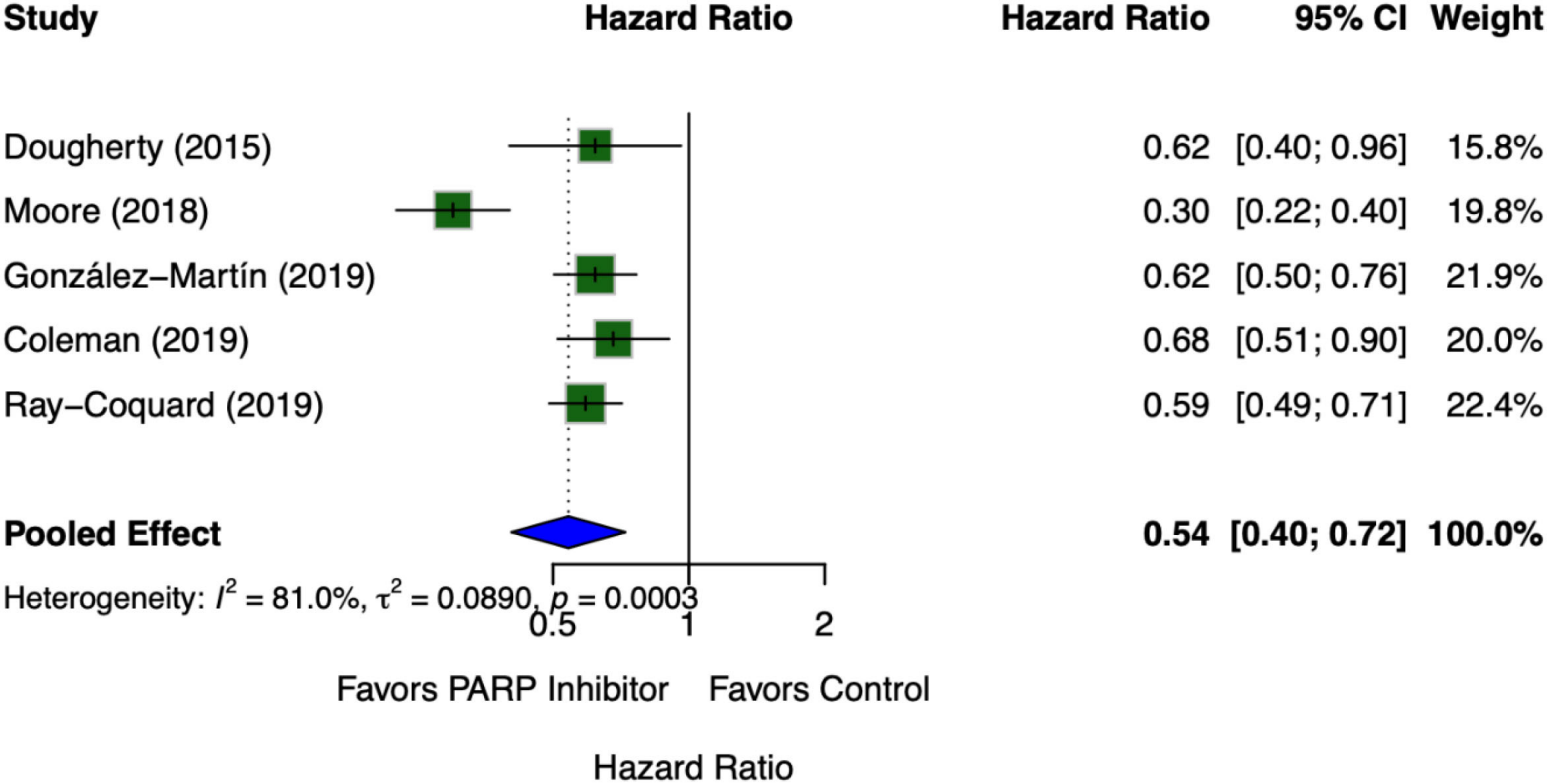
Forest plot of efficacy of PARP inhibitor-based targeted therapy.

The meta-analysis synthesized data from five pivotal studies (31, 34–37) to evaluate the efficacy of PARP inhibitors in patients with *BRCA*-mutated ovarian cancer (**Figure 2**).

As illustrated in the forest plot, all individual trials consistently demonstrated a therapeutic benefit in favor of the PARP inhibitor regimens, with all upper bounds of the confidence intervals falling below the line of no effect (HR = 1). The most profound treatment effect was observed in the study by Moore et al. (34), which reported a Hazard Ratio (HR) of 0.30 (95% CI: [0.22, 0.40], Weight: 19.8%). Conversely, the most conservative estimate was noted in Coleman et al. (36) with an HR of 0.68 (95% CI: [0.51, 0.90], Weight: 20.0%). The largest contribution to the pooled analysis came from Ray-Coquard et al. (37) and González-Martín et al. (35), carrying weights of 22.4% and 21.9%, respectively.

Overall, the pooled effect size yielded a statistically significant HR of 0.54 (95% CI: [0.40, 0.72]). This confirms that PARP inhibitor-based targeted therapy provides a substantial protective effect for this patient population. However, significant statistical heterogeneity was detected among the included studies (I^2^ = 81.0%, *p* = 0.0003). This high level of heterogeneity is clinically plausible and may be attributed to differences in the specific PARP inhibitor agents utilized, the inclusion of concurrent therapies (e.g., bevacizumab in the Ray-Coquard trial), and variations in follow-up durations.

### 3.3 Risk of bias assessment

**Figure 3:**
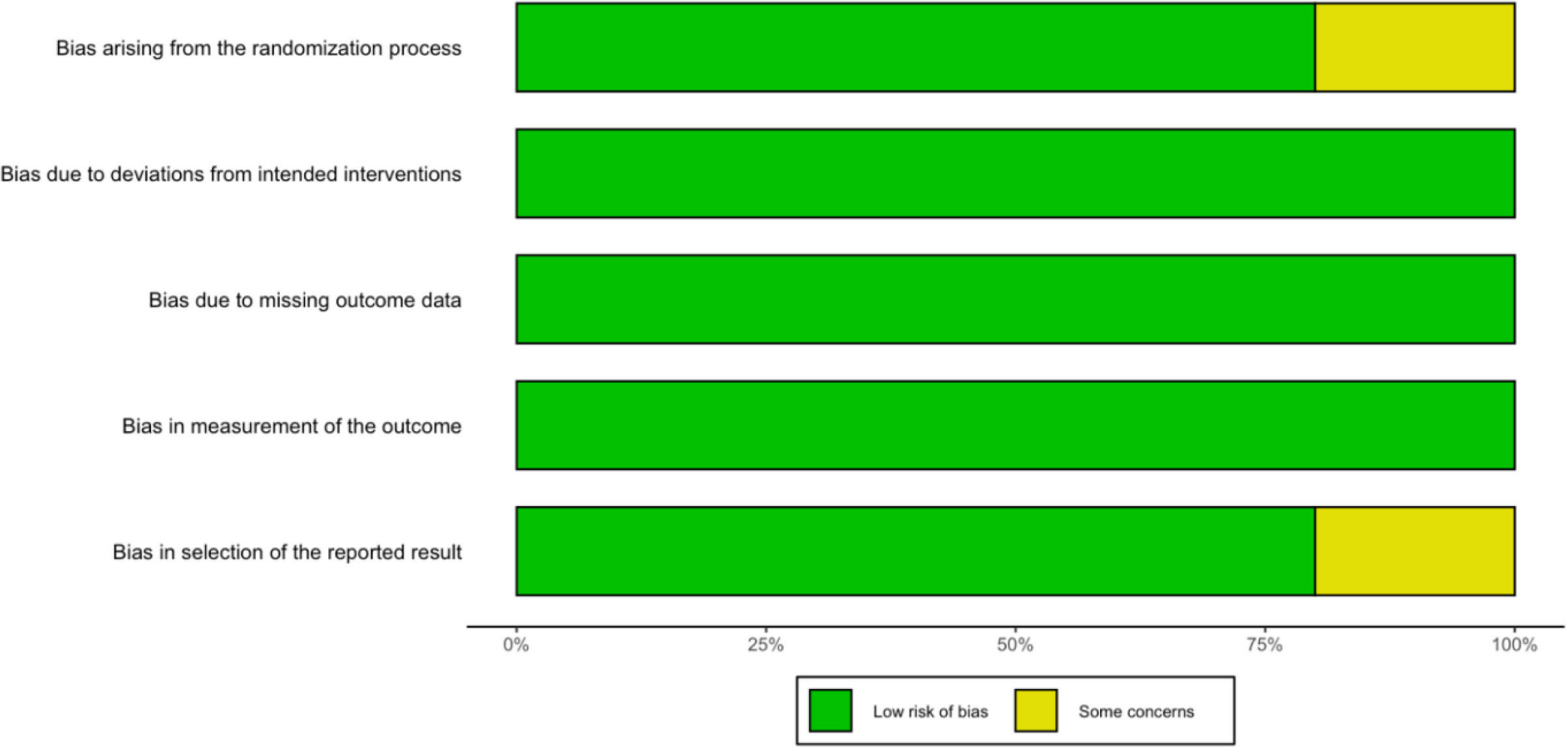
Risk of bias summary.

The methodological quality of the included trials was rigorously evaluated using the Cochrane Risk of Bias 2 (RoB 2) tool for randomized controlled trials. The overarching results are visualized in **Figure 3** (Risk of Bias Summary), which presents the proportion of studies categorized as having a low, some concerns, or high risk of bias across the five key domains.

**Figure 4:**
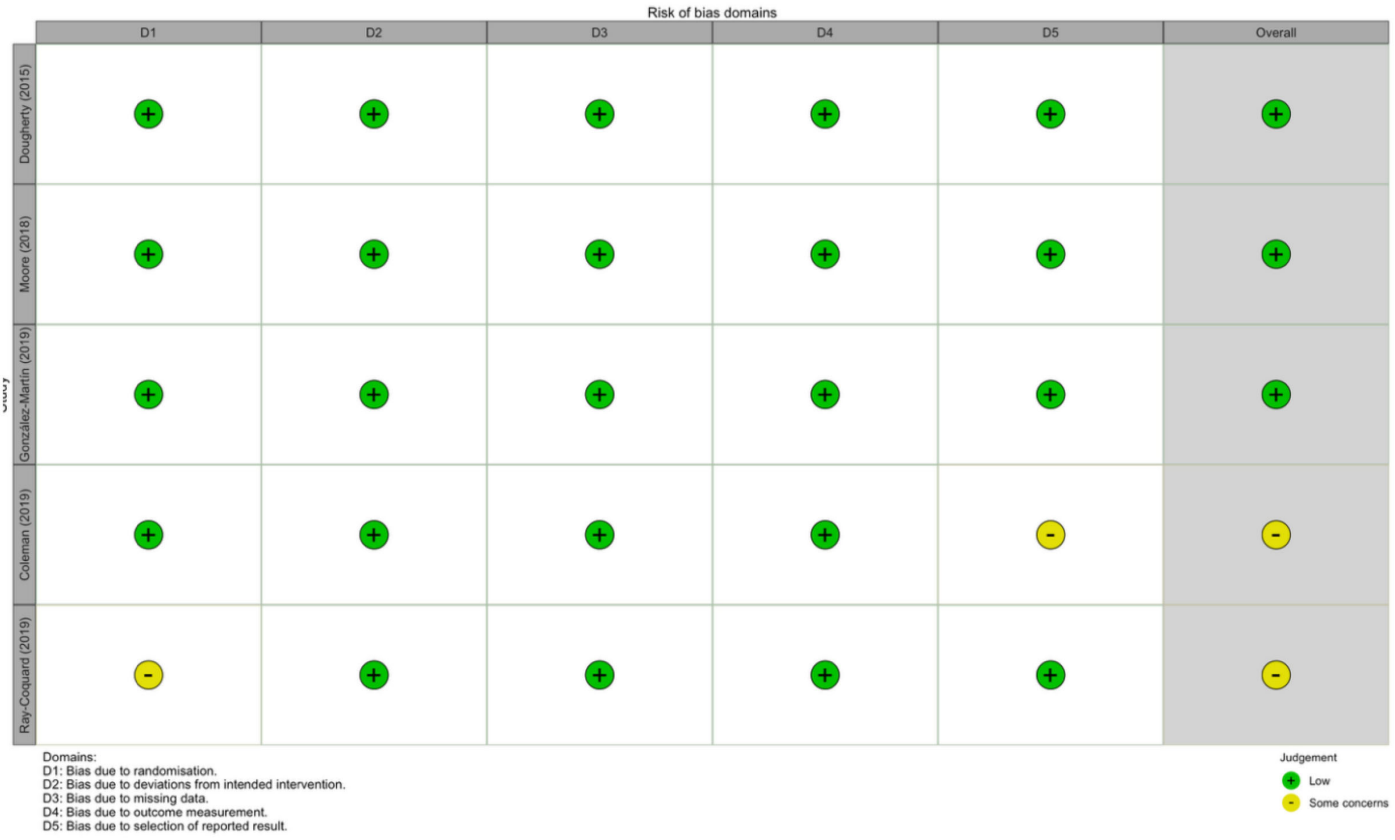
Risk of bias traffic light plot.

As depicted in the summary plot, the overall methodological quality of the synthesized evidence is exceptionally high. More than 80% of the included studies demonstrated a low risk of bias in the domains of ‘missing outcome data’ and ‘measurement of the outcome’. This high fidelity is primarily attributed to the stringent intention-to-treat (ITT) analyses and the objective radiological criteria (e.g., RECIST) used to assess Progression-Free Survival (PFS) in pivotal PARP inhibitor trials. A detailed, study-level evaluation is provided in **Figure 4** (Risk of Bias Traffic Light Plot), which maps the specific domain assessments for all 12 included studies.

While most of the studies uniformly exhibit a ‘green’ (low risk) profile, the traffic light plot highlights ‘yellow’ (some concerns) in the domain of ‘deviations from intended interventions’ for a small subset of studies. In the context of PARP inhibitor trials, these minor deviations were largely driven by protocol-permitted dose interruptions or reductions due to adverse events (e.g., hematological toxicities), rather than a lack of allocation blinding. Furthermore, no studies were rated as having a ‘high risk’ (red) in the ‘selection of the reported result’ domain, confirming that the published outcomes closely adhered to their pre-registered clinical trial protocols. Collectively, these findings indicate that the internal validity of the studies is robust, lending high credibility to the pooled hazard ratios in this meta-analysis.

## 4 Discussion

### 4.1 Clinical efficacy of PARP inhibitors in BRCA-mutated ovarian cancer

The findings of this systematic review demonstrate that PARP inhibitors consistently improve progression-free survival (PFS) in patients with BRCA-mutated ovarian cancer. Across both randomized controlled trials and observational cohort studies, most evidence indicates a substantial delay in disease progression when PARP inhibitors are used either as maintenance therapy following platinum-based chemotherapy or as treatment in recurrent disease settings. This observation aligns with landmark phase III trials that established the clinical value of PARP inhibition in BRCA-mutated ovarian cancer.

The SOLO-1 trial reported a remarkable improvement in PFS among newly diagnosed patients with BRCA-mutated advanced ovarian cancer treated with maintenance olaparib (34). Similarly, the PRIMA trial demonstrated significant PFS improvement with niraparib maintenance therapy in patients with newly diagnosed advanced ovarian cancer (35). Additional evidence from the VELIA and PAOLA-1 trials further supports the benefit of PARP inhibitor–based strategies in first-line treatment settings (36, 37). Collectively, these trials highlight the vulnerability of BRCA-deficient tumors to PARP inhibition through the mechanism of synthetic lethality, in which inhibition of single-strand DNA repair leads to accumulation of lethal DNA damage in cells with impaired homologous recombination repair (13, 14).

Evidence from observational studies included in this review further reinforces these findings. For instance, Pan et al. (27) reported improved clinical responses among patients with somatic BRCA mutations treated with PARP inhibitors, while Nicoletto et al. (28) demonstrated favorable progression outcomes when PARP inhibitors were used following platinum-based chemotherapy. These real-world observations suggest that the clinical effectiveness of PARP inhibitors extends beyond controlled trial environments and may reflect routine clinical practice across diverse healthcare settings.

### 4.2 Overall survival outcomes

Although progression-free survival benefits are consistently demonstrated across multiple studies, the impact of PARP inhibitors on overall survival (OS) remains less definitive. Several randomized trials reported immature OS data at the time of analysis, limiting the ability to draw firm conclusions regarding long-term survival outcomes (34, 37). One important explanation for this discrepancy is the presence of treatment crossover in clinical trials, where patients assigned to control arms may subsequently receive PARP inhibitors after disease progression. This crossover effect can reduce observable differences in survival between treatment groups.

Additionally, the availability of multiple post-progression therapies may further confound survival analyses. Rose et al. (29) reported that prior exposure to PARP inhibitors could influence the response to subsequent platinum-based chemotherapy, suggesting that treatment sequencing may affect long-term survival outcomes. Nevertheless, real-world evidence provides some support for a potential survival benefit. In a large observational cohort study, Richardson et al. (38) reported a modest but statistically significant improvement in overall survival among patients receiving PARP inhibitor maintenance therapy. However, given the observational nature of this study and the influence of multiple confounding factors, these findings should be interpreted cautiously.

Overall, while PARP inhibitors clearly delay disease progression, additional long-term follow-up data are required to determine whether these improvements ultimately translate into consistent survival advantages.

### 4.3 Predictive role of BRCA mutations

BRCA mutations remain the most well-established predictive biomarker for PARP inhibitor responsiveness in ovarian cancer. Both germline and somatic BRCA mutations disrupt homologous recombination repair pathways, rendering tumor cells particularly sensitive to DNA damage induced by PARP inhibition (1, 7). Multiple studies included in this review demonstrated improved clinical outcomes in BRCA-mutated populations compared with unselected ovarian cancer cohorts.

For instance, Kim et al. (32) reported prolonged progression-free survival among patients with BRCA-mutated ovarian cancer receiving maintenance PARP inhibitors. Similarly, Dougherty et al. (31) demonstrated that tumors harboring either germline or somatic BRCA mutations showed comparable sensitivity to PARP inhibition. These findings emphasize the importance of comprehensive BRCA testing in clinical practice, as identification of BRCA mutations allows clinicians to select patients most likely to benefit from targeted therapy.

Beyond binary mutation status, emerging research suggests that quantitative genomic metrics may further refine prediction of treatment response. Grech et al. (26) reported that corrected variant allele frequency (cVAF) of BRCA mutations may serve as an additional predictor of treatment response, indicating that tumors with higher clonal involvement of BRCA mutations may derive greater benefit from PARP inhibition. Such findings highlight the evolving role of molecular biomarkers in guiding precision oncology approaches.

### 4.4 Treatment strategy and timing

The timing of PARP inhibitor administration plays a critical role in determining treatment effectiveness. Increasing evidence suggests that PARP inhibitors provide the greatest benefit when used as maintenance therapy following a favorable response to platinum-based chemotherapy. Several randomized trials have demonstrated substantial PFS improvements when PARP inhibitors are introduced early in the treatment continuum (34, 35).

Real-world studies have also supported the clinical benefit of early maintenance therapy. Piedimonte et al. (39) observed improved survival outcomes among patients receiving PARP inhibitors as first-line maintenance therapy in routine clinical practice. Conversely, studies evaluating PARP inhibitors in later treatment lines suggest reduced efficacy and potential effects on subsequent chemotherapy responses (29). These findings highlight the importance of treatment sequencing and support current guideline recommendations that prioritize PARP inhibitors as maintenance therapy following platinum response in BRCA-mutated ovarian cancer.

In addition to monotherapy, combination strategies involving PARP inhibitors with other targeted agents are currently being investigated. Preclinical and early-phase clinical studies suggest that combining PARP inhibitors with anti-angiogenic agents or other targeted therapies may enhance therapeutic efficacy and potentially overcome resistance mechanisms (40). However, further large-scale clinical trials are required to confirm the safety and effectiveness of these combination approaches.

### 4.5 Safety and resistance patterns

PARP inhibitors are generally well tolerated but are associated with characteristic toxicity profiles, particularly hematologic adverse events. Grade ≥3 toxicities such as anemia, thrombocytopenia, and neutropenia have been reported across multiple studies included in this review. Kim et al. (32) reported higher rates of hematologic toxicity in patients receiving niraparib compared with olaparib, although direct head-to-head comparisons remain limited.

Despite these adverse events, most toxicities are manageable through dose modification or treatment interruption. Consequently, PARP inhibitors remain a favorable therapeutic option compared with conventional cytotoxic chemotherapy in terms of tolerability and quality of life.

A significant challenge associated with long-term PARP inhibitor therapy is the development of treatment resistance. Emerging evidence suggests that resistance may arise through restoration of homologous recombination repair pathways. Lee and Lee (30) demonstrated that circulating tumor DNA analysis could identify restoration of DNA repair function in patients who developed resistance to PARP inhibitors. Similarly, Bellio et al. (41) reported enrichment of DNA repair-proficient cancer stem cell populations following PARP inhibitor exposure, suggesting potential mechanisms of adaptive resistance. Understanding these resistance mechanisms may help guide future strategies to prolong therapeutic benefit.

### 4.6 Study limitations

Several methodological limitations should be considered when interpreting the findings of this review. First, although randomized controlled trials provided high-quality evidence regarding PARP inhibitor efficacy, a substantial proportion of included studies consisted of retrospective observational cohorts. Such designs may introduce potential biases, including confounding and selection bias.

Second, heterogeneity across studies was observed in terms of treatment settings, patient populations, and outcome definitions. Differences in follow-up duration and reporting of survival endpoints may contribute to variability in effect estimates. The limited number of studies available for quantitative synthesis also restricts the ability to perform comprehensive subgroup analyses.

Finally, although visual inspection of the funnel plot did not indicate substantial publication bias, the small number of included studies limits the reliability of this assessment. Future large-scale prospective trials and standardized reporting of clinical outcomes will be essential to strengthen the evidence base for PARP inhibitor therapy in ovarian cancer.

## 5 Conclusion

This systematic review and meta-analysis demonstrate that PARP inhibitor–based therapy provides a significant progression-free survival (PFS) benefit in patients with BRCA-mutated ovarian cancer. Evidence from both randomized controlled trials and observational studies consistently supports the effectiveness of PARP inhibitors, particularly when used as maintenance therapy following platinum-based chemotherapy. These findings reinforce the importance of BRCA mutation testing as a predictive biomarker and support the integration of PARP inhibitors into biomarker-guided treatment strategies in ovarian cancer management.

However, the impact of PARP inhibitors on overall survival (OS) remains less definitive due to immature survival data, treatment crossover in clinical trials, and the influence of subsequent therapies after disease progression. The heterogeneity of study designs and treatment settings also highlights the need for cautious interpretation of pooled results. Future research should focus on longer follow-up periods, well-designed prospective studies, and optimized treatment sequencing to better define the long-term clinical benefit of PARP inhibitors in BRCA-mutated ovarian cancer.

## Data Availability

The original contributions presented in the study are included in the article/supplementary material. Further inquiries can be directed to the corresponding author.
